# Implementation and Performance of a GPS/INS Tightly Coupled Assisted PLL Architecture Using MEMS Inertial Sensors

**DOI:** 10.3390/s140203768

**Published:** 2014-02-24

**Authors:** Youssef Tawk, Phillip Tomé, Cyril Botteron, Yannick Stebler, Pierre-André Farine

**Affiliations:** 1 École Polytechnique Fédérale de Lausanne, Institute of Microengineering (IMT), Electronics and Signal Processing Laboratory, Neuchâtel, Switzerland; E-Mails: phillip.tome@epfl.ch (P.T.); cyril.botteron@epfl.ch (C.B.); pierre-andre.farine@epfl.ch (P.-A.F.); 2 École Polytechnique Fédérale de Lausanne, Geodetic Engineering Laboratory, Lausanne, Switzerland; E-Mail: yannick.stebler@epfl.ch

**Keywords:** GPS, INS, MEMS inertial sensor, tight coupling, extended kalman filter, tracking, acquisition, navigation

## Abstract

The use of global navigation satellite system receivers for navigation still presents many challenges in urban canyon and indoor environments, where satellite availability is typically reduced and received signals are attenuated. To improve the navigation performance in such environments, several enhancement methods can be implemented. For instance, external aid provided through coupling with other sensors has proven to contribute substantially to enhancing navigation performance and robustness. Within this context, coupling a very simple GPS receiver with an Inertial Navigation System (INS) based on low-cost micro-electro-mechanical systems (MEMS) inertial sensors is considered in this paper. In particular, we propose a GPS/INS Tightly Coupled Assisted PLL (TCAPLL) architecture, and present most of the associated challenges that need to be addressed when dealing with very-low-performance MEMS inertial sensors. In addition, we propose a data monitoring system in charge of checking the quality of the measurement flow in the architecture. The implementation of the TCAPLL is discussed in detail, and its performance under different scenarios is assessed. Finally, the architecture is evaluated through a test campaign using a vehicle that is driven in urban environments, with the purpose of highlighting the pros and cons of combining MEMS inertial sensors with GPS over GPS alone.

## 1. Introduction

The advantages of combining satellite-based navigation systems with inertial navigation systems (INS) have long been recognized. Due to the various errors induced by the space segment, signal propagation, receiver technology or user environment, the accuracy of global navigation satellite system (GNSS) receivers is not sufficient to address the requirements of all applications in geodesy and navigation. Therefore, many navigation or surveying systems use hybrid GNSS/INS technologies to combine the short time stability of the inertial sensors with the long time stability, yet noisy behavior, of GNSS receivers. Depending on the integration scheme and the performance of the inertial sensors, a variety of advantages in accuracy, availability, and integrity can be achieved [1]. The three possible basic GNSS/INS integration schemes are loose, tight and ultra-tight (or deep) integration [2]. In the loose approach, the position and velocity from both systems are integrated to create a third blended navigation solution. In the tight approach, GNSS pseudoranges and Doppler are blended with INS accelerometer and gyro measurements to generate a single blended navigation solution. In the ultra-tight approach, integration occurs at the GNSS tracking loops which are controlled by the blended navigation filter [3]. The GNSS receiver in this case is no longer an independent navigator since its operation is also partly dependent on INS information. The potential benefits of the deep integration are achieved at the expense of a significant increase in complexity, computational load, and tight time synchronization [4]. It is worth mentioning that there is not a standardized definition for the types of GNSS/INS integrations. While some papers refers to ‘ultra-tight’ or ‘deep’ integration when there is a feed-forward component from the navigation filter to the GNSS tracking loops, other papers (such as this one) refer to such setups as ‘tight’ coupling and use the terms ‘ultra-tight’ or ‘deep’ only when the tracking loops' numerically controlled oscillators (NCOs) are fully controlled by the blended navigation filter in a vector tracking approach. More details about the different naming conventions can be found in [5]. The theory and measurement models of such ultra-tight GNSS/INS schemes have already been provided in many research works. For instance, in [6–8], the authors present analytical and simulation results under specific scenarios, but they do not provide fields experiment results.

In recent years, micro-electro-mechanical systems (MEMS) inertial sensors have opened the door to a wealth of new commercial applications and are present in various items that are used on a daily basis (e.g., most smart phones on the market already include at least an accelerometer, a gyroscope or a magnetometer). Apart from size reduction, MEMS technology has offered many benefits such as batch production, cost and power reductions and design flexibility, within limits. However, the reduction in size of the sensing elements creates challenges for attaining good performance. Several papers have already discussed the integration of MEMS inertial sensors with GPS. For example, in [9], the authors described the design, operation and performance of an integrated GPS/MEMS inertial navigation package, without using the inertial measurements to aid the GPS tracking loops. In [10], the author proposes a tightly coupled MEMS INS/GPS integration with INS-aided receiver tracking loops, but he does not discuss the quality of aiding, especially when GPS outages occur or when fewer satellites are tracked. The impact on the GPS tracking loops was partially addressed and no assessment was made of the benefits of such integration on re-acquisition. Also, in [11], the author proposes a tight integration with loop aiding using a GPS/reduced inertial measurement unit (IMU) coupling and an adaptive loop filter bandwidth. However, the impact on re-acquisition and the GPS outage limitations are not assessed. In addition to these papers, other works discuss the integration of MEMS inertial sensors with a GPS receiver for various applications (see e.g., [12,13]).

Within this context, we propose in this paper a GPS/INS tightly coupled assisted PLL (TCAPLL) architecture aiming to reveal more precisely the advantages, limitations and drawbacks that can be encountered when using MEMS inertial sensors for land navigation. The architecture is implemented using an extended Kalman filter (EKF) that combines pseudoranges and Doppler measurements from a very simple GPS receiver with the INS measurements to obtain a position, velocity and attitude solution, and later send a feed-forward aiding component to the PLL tracking loops to compensate for the dynamics and GPS receiver clock frequency error. A monitoring system is also implemented to check the quality of the aiding information to ensure robust tracking especially when few satellites are being tracked. The performance of the architecture is evaluated by simulations under different scenarios assuming a commercial off-the-shelf low-quality MEMS inertial sensor. The assessment is made in terms of navigation accuracy, tracking robustness, sensitivity and re-acquisition time. Finally, the new architecture is tested in a measurement campaign consisting of a test vehicle driven in an urban canyon environment and mounted with a GNSS antenna connected to a front-end, a MEMS IMU, and a navigation grade INS for the reference trajectory. Measurements from these three sensors are gathered and post-processed to highlight the advantages of the TCAPLL architecture over a stand-alone GPS solution.

## 2. TCAPLL Architecture

**Figure 1.**
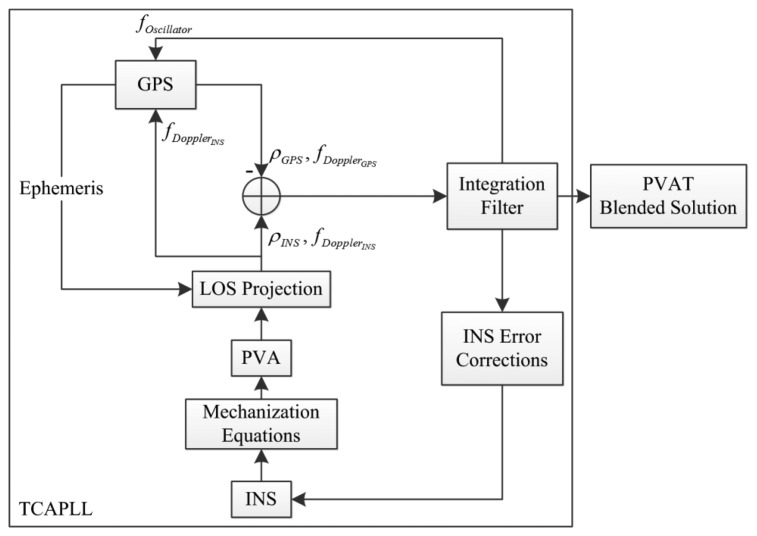
TCAPLL architecture.

The TCAPLL architecture is shown in Figure 1 where the four main blocks of the architecture can be ideied as the GPS, the INS the integration filter and the data monitoring system. The measurement processing starts by running the GPS receiver alone and, once the ephemeris and a position, velocity and time (PVT) solution are obtained, the INS can be initialized and synchronized with the GPS time. Next, the position, velocity and attitude (PVA) solution from INS is projected into LOS to obtain INS ranges and Doppler frequencies which are combined with the raw pseudoranges and Doppler measurements from the GPS tracking loops to form the input of the centralized integration filter. The filter directly accepts their differences to compute the error corrections which are used to update the final position, velocity, attitude and time (PVAT) solution and the INS predicted errors, *i.e.*, bias drifts and scale factors. The Doppler frequency predicted by the INS is added to the GPS oscillator frequency drift estimated by the integration filter to form a feed-forward component to assist the GPS PLL tracking loops. Note that the predicted INS Doppler frequency is taken directly from the output of the LOS projection block instead of the integration filter. The reason for this is that the integration filter update frequency is much lower than the INS prediction frequency. Therefore, to keep the GPS tracking loops up to date with the latest Doppler frequency, the INS output is taken and the corresponding INS Doppler is corrected every time the integration filter is updated. As a result, because the PLL tracking loops have an external aiding frequency, the dynamics and the receiver oscillator clock errors do not need to be tracked any more by the PLL loop filter and only the PLL thermal noise and the error of the external aiding component should be accounted for.

As we intend to use low-quality MEMS inertial sensors, which are characterized by high integration drifts, the measurements from the INS have to be continuously monitored in order to validate the computed navigation solution, and to select when to enable and disable the feed-forward component. This timing depends on two factors: the INS sensor quality and the integration filter. Therefore, a monitoring system responsible for checking the quality of the INS measurements is implemented as part of the TCAPLL architecture in order to ensure robust tracking and accurate navigation. In addition, the data monitoring system observes the GPS tracking loops to check the visibility of the tracked satellites and to validate their corresponding raw measurements. The design of each of the TCAPLL blocks is described in the following sections, but first the model of the errors of the MEMS-based IMU used in the evaluation of the TCAPLL is presented.

### 2.1. IMU MEMS Error Modeling

Generally, IMU sensor errors can be modeled by stochastic processes. Depending on the nature of the error (e.g., random or systematic, correlated over time or uncorrelated), different types of stochastic processes such as random constant, random walk, or first-order Gauss-Markov (GM) can be used to model it. Typical remaining uncompensated errors (after calibration) of an accelerometer and gyroscope include a turn-on to turn-on residual bias, axis misalignment, a residual scale-factor error, correlated noise and white noise. For high-grade IMUs, the manufacturer calibrates the INS extensively and compensates for these errors in its processor; therefore only small random errors remain. However, for low-cost MEMS inertial sensors, deterministic errors persist and can be roughly estimated by lab calibrations or manufacturer specifications [14]. Therefore, a solution to this problem is to include these errors as part of the functional model and to estimate them in a Kalman filter. In this paper, the MEMS sensor errors on each axis of the accelerometer and the gyroscope are modeled as [14]:

(1) $$\delta \omega_{ib}^{b}=b_{g}+\delta b_{g}+\omega_{b}SF_{g}+\eta_{g}$$

(2) $$\delta f_{ib}^{b}=b_{a}+\delta b_{a}+f_{b}SF_{a}+\eta_{a}$$

where $\delta \omega_{ib}^{b}$ and $\delta f_{ib}^{b}$ are the total angular rate and force error in the body frame, *η_g_* and *η_a_* are white noise errors with spectral densities *σ_g_* and *σ_a_*, respectively, and *b_g_* and *b_a_* are turn-on-to-turn-on biases, and are modeled as a random constant process, given by:

(3) $${\dot{b}}_{i}=0$$

where ‘*i*’ stands for ‘*g*’ for gyroscope or ‘*a*’ for accelerometer. *δb_g_*, *δb_a_*, *SF_g_*, and *SF_a_* are the bias drifts and scale factor errors, and are modeled as first-order GM processes, given by:

(4) $$\delta {\dot{b}}_{i}=-\beta_{d_{i}}\delta b_{i}+\eta_{d_{i}}$$

(5) $$S{Ḟ}_{i}=-\beta_{SF_{i}}SF_{i}+\eta_{SF_{i}}$$

where *β_d_i__* and *β_SF_i__* are the inverse of the process correlation times, and *η_d_i__* and *η_SF_i__* are the GM process driving noises with spectral densities *σ_δb_i__* and *σ_SF_i__* , respectively.

### 2.2. Integration Filter

The GPS/INS coupling is a non-linear system, and as seen in the previous section, most of the MEMS INS errors have non-Gaussian noise. Therefore an EKF is used as the integration filter in the TCAPLL architecture. The EKF applies the Taylor series expansion for the non-linear system and observation equations, and takes the first-order terms to apply the well-developed linear Kalman filter theory, where the probability density function (PDF) is approximated by a Gaussian model [15]. This approach is adopted to derive a set of linear differential equations that define the INS error states. Details can be found in [16]. The state vector of the TCAPLL EKF is composed of 29 states and is given as:

(6) δẋ=Fδx+Gw

where **F** is the dynamic matrix, **G** is the noise matrix, **w** is the Gaussian white noise vector and *δ****x*** is the augmented error state vector, expressed as:

(7) $${\left(\delta \text{x}\right)}_{29\times 1}={\left[\begin{array}{lllllllllll} \delta {\text{r}}_{1\times 3}^{l} & \delta {\text{v}}_{1\times 3}^{l} & \delta {\text{a}}_{1\times 3}^{l} & b_{a1\times 3} & \delta b_{a1\times 3} & SF_{a1\times 3} & b_{g1\times 3} & \delta b_{g1\times 3} & SF_{g1\times 3} & \delta t_{b} & \delta t_{d} \end{array}\right]}^{T}$$

where $\delta {\text{r}}_{1\times 3}^{l}$, $\delta {\text{v}}_{1\times 3}^{l}$, and $\delta {\text{a}}_{1\times 3}^{l}$ are the position, velocity and attitude errors in the local-level frame, which is chosen to be the ENU (East, North, Up) in this paper. *δt_b_* and *δt_d_* are the GPS receiver oscillator bias and drift. Their differential equations are written as [17]:

(8) $$\delta {ṫ}_{b}=\delta t_{d}+\eta_{b}^{\text{osc}}$$

(9) $$\delta {ṫ}_{d}=\eta_{d}^{\text{osc}}$$

where $\eta_{b}^{\text{osc}}$ and $\eta_{d}^{\text{osc}}$ are Gaussian-distributed white noises. As stated before, the difference between the raw pseudoranges and Doppler measurements from the GPS tracking loops, together with the INS ranges and Doppler frequencies, form the input of the centralized integration filter. The derivation of the analytical parameters of the state vector and the measurement model of the TCAPLL integration filter can be found in [18]. It is important to mention that the well-known INS mechanization equations are used to convert the output of the IMU into a PVA solution and further propagate it in time. In the TCAPLL architecture, we used quaternions for attitude representation and propagation.

### 2.3. GPS Assisted PLL

**Figure 2.**
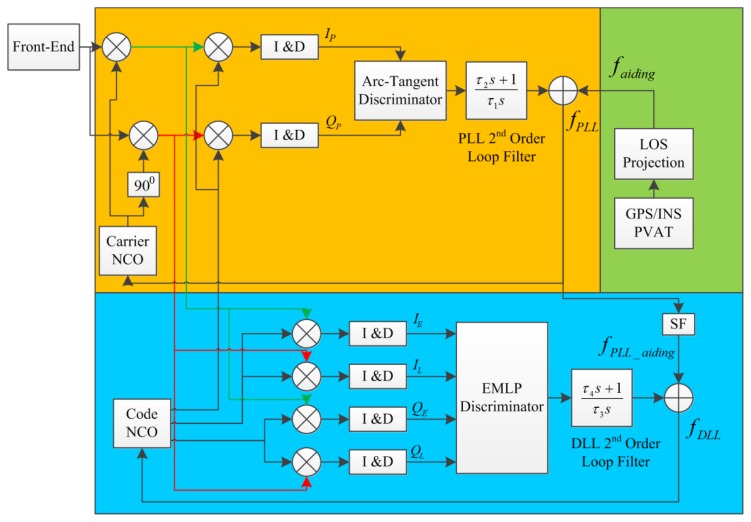
GPS-assisted PLL and DLL loops.

The feed-forward component to assist the GPS PLL of satellite ‘*k*’ is computed as:

(10) $$f_{\text{aiding}}^{k}=f_{{\text{Doppler}}_{\text{INS}}}^{k}+f_{\text{oscillator}}=f_{{\text{Doppler}}_{\text{INS}}}^{k}+\delta t_{d}/\lambda_{L1}$$

where $f_{{\text{Doppler}}_{\text{INS}}}^{k}$ is the Doppler frequency of satellite ‘*k*’ computed from the INS navigation solution and *λ_L_*_1_ is the wavelength of the GPS L1 signal. This component slightly modifies the typical design of the PLL. Figure 2 shows the GPS PLL and DLL tracking loops used in the TCAPLL architecture.

**Figure 3.**
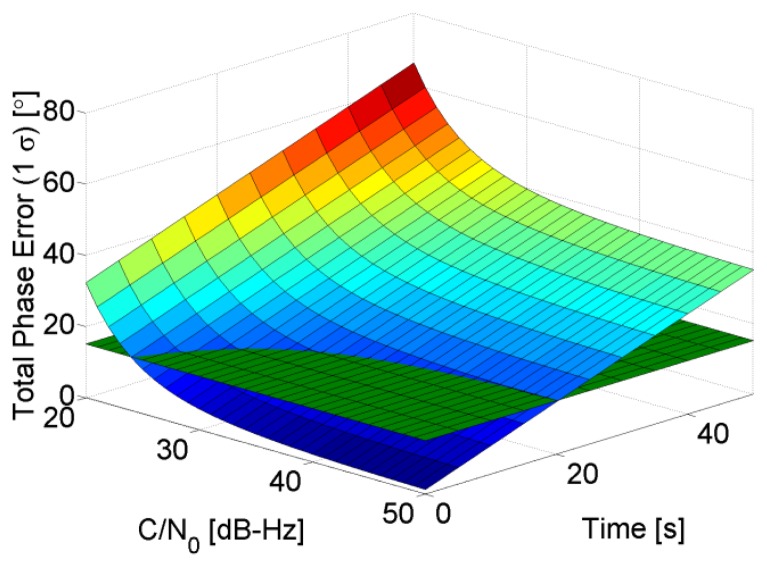
Assisted PLL phase error when *B_n_* = 5 Hz, *T_int_* = 1 ms, $\delta f_{ib}^{b}=0.12\;{\text{m}/\text{s}}^{2}$, and $\delta \omega_{ib}^{b}=2,000\;\text{deg}/\text{h}$.

Once the feed-forward component is enabled, the PLL and DLL are reset, and the feed-forward component is added to the output of the PLL loop filter. The input to the carrier NCO is expressed as:

(11) $$f_{\text{PLL}}=f_{\text{aiding}}+f_{{\text{PLL}}_{\text{noise}}}$$

where $f_{{\text{PLL}}_{\text{noise}}}$ is the thermal noise of the PLL. The input to the carrier NCO is also used as assistance to the DLL by multiplying it with a scale factor (SF) and adding it to the output of the DLL filter. The input to the code NCO is expressed as:

(12) $$f_{\text{DLL}}=\frac{f_{\text{PLL}}}{\text{SF}}+f_{{\text{DLL}}_{\text{noise}}}=f_{\text{PLL}\_\text{aiding}}+f_{{\text{DLL}}_{\text{noise}}}$$

where $f_{{\text{DLL}}_{\text{noise}}}$ is the thermal noise frequency of the DLL, and SF is equal to 1,540 (*i.e.*, *f_L_*_1_/*f_C/A_*). As we intend to use low-quality MEMS inertial sensors for the INS, it is important to evaluate the quality of aiding that can be expected from such sensors and their impact on the PLL loops. In order to do so, we need first to derive what is the error tolerable by the assisted PLL loops. In the presence of assistance, the 1-sigma rule of thumb PLL tracking threshold is expressed as:

(13) $$\sigma_{\text{PLL}}=\sqrt{\sigma_{\text{tPLL}}^{2}+\sigma_{\upsilon }^{2}+\sigma_{A}^{2}}+\frac{e_{f-f}}{3}\leq 15{}^{\circ}$$

where *σ_tPLL_* is the thermal noise jitter, *σ_υ_* is the vibration-induced oscillator jitter, and *σ_A_* is the Allan-variance-induced oscillator jitter. Their expressions can be found in [17]. *e_f_*_−_*_f_* is the error induced from the feed-forward component, which is dependent on the error sources of the IMU and can be highly correlated in time. In the case of INS assistance with no error corrections, *i.e.*, the sensor measurements are not corrected by the integration filter, *e_f_*_−_*_f_* (restricted to the horizontal plane) is expressed as [19]:

(14) $$e_{f-f}=\frac{0.2809}{B_{n}^{2}}\left(\delta f_{ib}^{b}\cos\left(\omega_{s}t\right)+\delta \omega_{ib}^{b}g\frac{\sin\left(\omega_{s}t\right)}{\omega_{s}}\right)$$

where g is the acceleration due to gravity, *ω_s_* is the Schuler frequency, and *t* is time. It is clear that with assistance, the PLL dynamic stress error no longer depends solely on the dynamics of the receiver, but also on time, because it depends on the inertial solution, which degrades over time. For example, let us assume that the INS has low-quality IMU having acceleration and angular velocity drifts of 0.12 m/s^2^ and 2,000 deg/h respectively. In this case the aiding component coming from the INS will drift in time and a 2nd order PLL (which is used typically in aided tracking loops [20]) with a bandwidth of 5 Hz and integration time of 1 ms will not be able to compensate the drift and according to Figure 3, it will lose lock after roughly 20 s. This means that MEMS inertial sensors cannot be used directly to assist a PLL. Hence error corrections through an EKF are mandatory to meet the PLL support requirements. In this case the assistance error depends not only on time, but also on the filter states' uncertainty, and in particular on the oscillator drift and estimated velocity which is used to compute the Doppler aiding.

**Figure 4.**
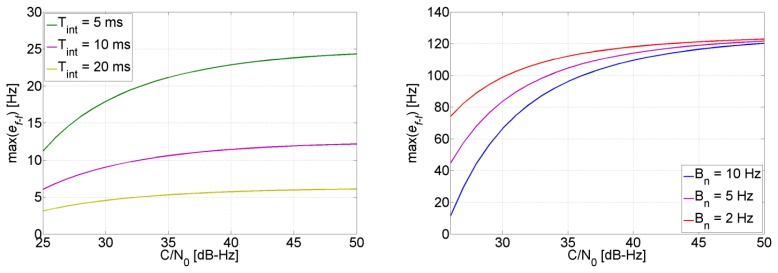
PLL tolerable assistance frequency error when *T_int_* = 1 ms (**left**) and *B_n_* = 5 Hz (**right**).

Therefore, taking into consideration that satellite velocity errors are corrected by the ephemeris, and that the LOS vector change is negligible, *e_f_*_−_*_f_* can be written as:

(15) $$e_{f-f}=3\sigma_{f_{\text{aiding}}}=3\sqrt{\sigma_{f_{{\text{Doppler}}_{\text{INS}}}}^{2}+\sigma_{f_{\text{Oscillator}}}^{2}}$$

where $\sigma_{f_{\text{Oscillator}}}^{2}$ can be taken directly from the EKF covariance matrix, and $\sigma_{f_{{\text{Doppler}}_{\text{INS}}}}^{2}$ for the satellite *S* is computed as [10]:

(16) $$\sigma_{f_{{\text{Doppler}}_{\text{INS}}}}^{2}=\frac{{\text{LOS}}_{S}P_{\text{v}}{\text{LOS}}_{S}^{T}}{\lambda_{L1}^{2}}$$

where *LOS_K_* is the LOS vector projection between the receiver and satellite *S* in the ECEF frame. **P_v_** is the covariance matrix of the computed velocity. It is important to note that there is no analytical model for **P_v_** because it depends on several factors such as the dynamics of the receiver, and the GPS observables that update the covariance matrix. Therefore it will be extracted from the TCAPLL EKF covariance matrix throughout the receiver trajectory. Using Equation (13), the resulting theoretical maximum allowable error by the PLL to maintain tracking is plotted in Figure 4 for different loop bandwidths and integration times (Practically, values that are close to, but not above, these maximums can be obtained). As it will be clarified in the next section, this maximum is taken as the reference in the monitoring system to enable and disable the feed-forward component.

## 2.4. Monitoring System

**Figure 5.**
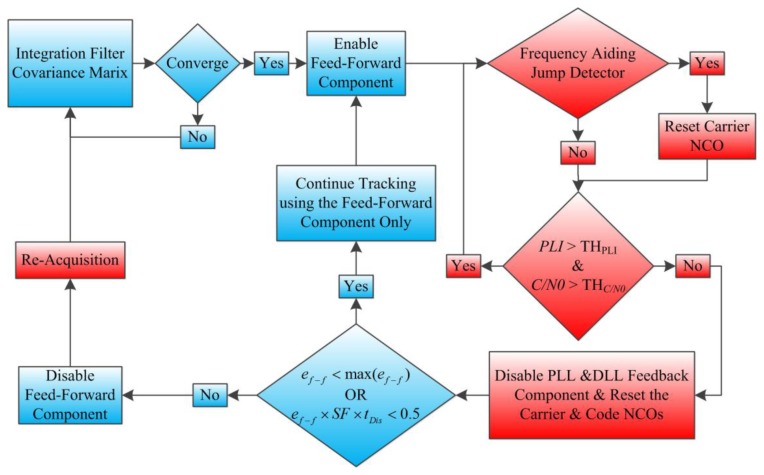
TCAPLL monitoring system implemented on each channel of the GPS receiver.

Low-cost MEMS inertial sensors are not well suited to be used directly as assistance to the GPS PLL loops. Therefore in addition to the error corrections that are provided continuously from the integration filter, a data monitoring system is implemented as part of the TCAPLL architecture in order to check the quality of the feed-forward component and the measurements from the GPS. The overall monitoring system, implemented on each GPS receiver channel, is shown in Figure 5, where the blue blocks correspond to the INS and the integration filter, and the red blocks correspond to the GPS receiver.

The data monitoring system first looks at the covariance matrix of the integration filter **P**. If it converges, the feed-forward component is enabled, *i.e.*, interrupter C in Figure 5 is closed, and the PLL starts to receive assistance. The convergence criteria can take into consideration different parameters of the filter states. In the TCAPLL architecture, we selected the geometry dilution of precision (GDOP) parameter by computing the uncertainty of the position and clock bias from the state covariance matrix and comparing it to a certain error threshold:

(17) $$\text{GDOP}=\sqrt{\sigma_{r_{x}}^{2}+\sigma_{r_{y}}^{2}+\sigma_{r_{z}}^{2}+\sigma_{\delta t}^{2}}\leq e_{\text{GDOP}}$$

Where *σ_r_x__* · *σ_r_y__* and *σ_r_z__* are the uncertainty of the position, *σ_δ_t__* is the uncertainty of the clock bias, and *e_GDOP_* is the error threshold. The latter depends on the quality of the MEMS IMU sensors, the GPS clock receiver and the targeted accuracy requirements, and typically it can take a value between 2 and 5. In the TCAPLL evaluation, the value of *e_GDOP_* is set to 3.

Once the feed-forward component is enabled, a jump detection is performed where the aiding frequency component of satellite *S* is compared to its previous value. If the difference is higher than the corresponding maximum tolerable frequency of the PLL loop filter, then the carrier NCO is reset. This can be described as:

(18) $${\left[{\text{cond}}_{\text{jump}}\right]}_{S}={\left[f_{\text{aiding}}\left(t_{k}\right)-f_{\text{aiding}}\left(t_{k-1}\right)\right]}_{S}<{\left[\max\left(e_{f-f}\right)\right]}_{S}$$

where [max(*e_f_*_−_*_f_*)]*_S_* can be obtained using Equation (13). Next, a test is performed on the GPS tracking loops to check whether satellite *S* is visible by computing the Phase Lock Indicator (PLI) and the *C*/*N*_0_ on its corresponding channel, and comparing them to two thresholds:

(19) $${\text{cond}}_{\text{track}}=\left[PLI_{S}>TH_{\text{PLI}}\right]\;\text{AND}\;\left[{\left[C/N_{0}\right]}_{S}>TH_{C/N_{0}}\right]$$

where *TH_PLI_* is the PLI threshold, which is selected to be 0.7 in this paper, and *TH*_*C/N*_0__ is the *C/N_0_* threshold, which depends on the integration time and PLL filter bandwidth [17]. If *cond_track_* is true, the tracking continues normally. If not, then the satellite is declared non-visible, and the feedback components from the PLL and DLL loop filters are disabled, *i.e.*, interrupters A and B in Figure 5 are opened. At this moment, a condition related to the frequency aiding uncertainty is checked:

(20) $${\text{cond}}_{e_{f-f}}=\left[{\left[e_{f-f}\right]}_{S}<\max\left(e_{f-f}\right)\right]\;\text{OR}\;\left[{\left[e_{f-f}\right]}_{S}\times {\left[t_{\text{Dis}}\right]}_{S}/\text{SF}<0.5\right]$$

The left condition of Equation (20) ensures that the aiding frequency maintains a carrier frequency error less than the maximum tolerable error of the PLL. The right condition of Equation (20) is related to the DLL loop filter and ensures that, as long as the satellite is non-visible during the disabling time of the feedback component *t_Dis_*, the aiding frequency maintains a code phase error of less than 0.5 chips. If these two conditions are satisfied, tracking continues, relying solely on the feed-forward component, which will allow the PLL and DLL of a tracking channel to re-track a satellite once it is visible again, without the need for re-acquisition. If not, the feed-forward component is disabled, and re-acquisition starts with a frequency search span equal to ±*e_f_*_−_*_f_*.

## 3. Simulation Testing Platform

**Figure 6.**
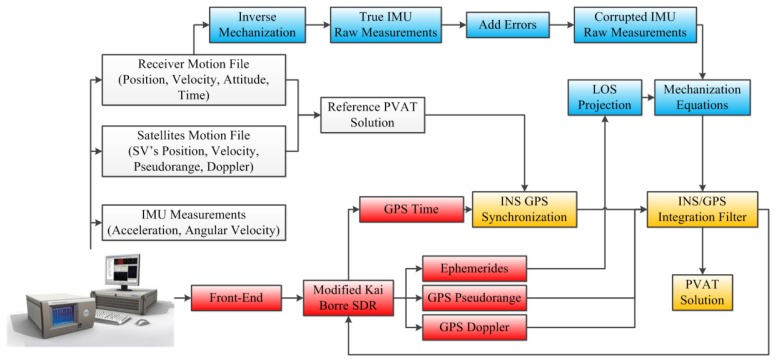
TCAPLL simulation testing platform.

The TCAPLL architecture is implemented mainly in Matlab and partially in the C language. The overall testing platform is shown in Figure 6. The Spirent GSS8000 simulator [21,22] is used to generate the GPS L1 signal and the scenario for the trajectory. The latter is obtained by driving a car mounted with a GPS receiver in Braunschweiger, Germany, and then the obtained raw GPS data are used in the Spirent simulator to create the trajectory. The simulator also provides a reference PVAT solution that is used first to synchronize the GPS receiver and the INS, and second to derive true acceleration and angular velocity measurements through an inverse mechanization. The true IMU measurements are corrupted later by different types of stochastic errors as described in Section 3.1 to obtain corrupted IMU raw measurements. By following this process, different types of sensors, in particular MEMS inertial sensors can be simulated. The characteristics of the errors used in the simulations are shown in Table 1 and represents typical error characteristics of COTS low quality MEMS inertial sensor [23–25].

For the GPS receiver, the Kai Borre software defined radio is used [26], with major modifications on the tracking loops, which have been changed from sequential channel tracking to parallel channel tracking, with the inclusion of the assistance for the PLL and DLL-assisted PLL. The number of channels is kept at eight, and the PLL and DLL discriminators used are the normalized Arc-Tangent and the Non-Coherent-Early-Minus-Late-Power. 2nd order PLL and DLL filters are implemented with bandwidths varying between 4 and 14 Hz for the PLL, and 1 and 2 Hz for the DLL. The early-late chip spacing is 1 chip, and two integration times are tested: 1 and 5 ms. The mechanization equations and the EKF were implemented in Matlab, where the INS frequency was set to 200 Hz and the GPS update rate to 1 Hz.

## 4. Simulation Results

To assess the performance and the advantages of integrating a MEMS inertial sensor with a GPS receiver, the TCAPLL architecture is tested under different scenarios. We start by analyzing the behavior under normal conditions, *i.e.*, open sky and seven visible satellite vehicles (SVs). Then, we degrade navigation conditions by decreasing the number of visible satellites to four, then three, and then zero (*i.e.*, GPS total outage). Under all these conditions, different PLL loop bandwidths and integration times are tested. The assessment takes into consideration navigation accuracy, tracking robustness, sensitivity and quality of the feed-forward component.

### 4.1. Open Sky with Seven SVs

**Figure 7.**
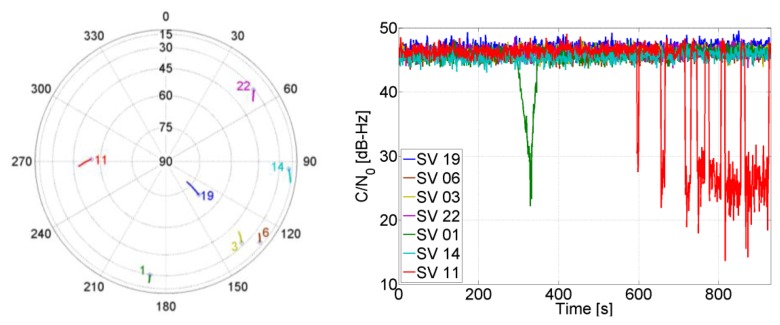
Sky plot during the simulated trajectory (**left**) and received power from satellites (**right**).

The sky plot during the simulated trajectory is shown in Figure 7, alongside the corresponding received power from the satellites. At the 300 s, the power of SV 01 starts to decrease with a step of 1 dB/s for a 25 s and then it increases again to reach its nominal value. From 600 s until 900 s, partial outages of SV 11 for different durations start to occur.

**Figure 8.**
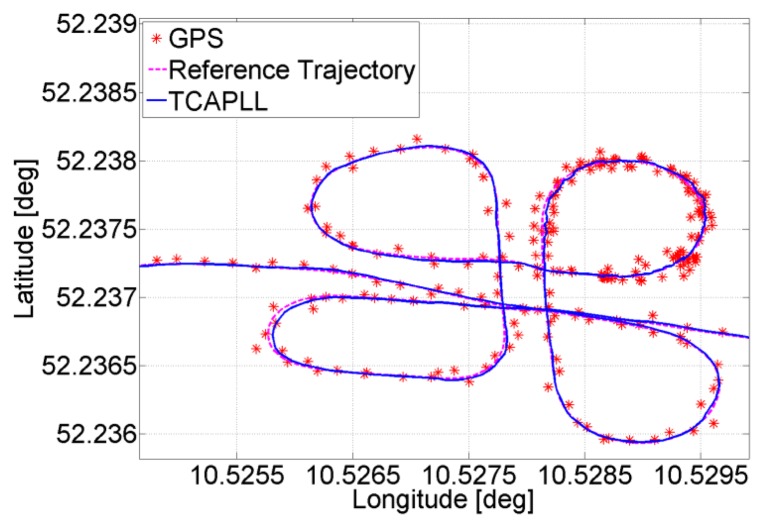
Comparison of the position solution of the TCAPLL and the stand-alone GPS.

The position solution of a small part of the trajectory is given in Figure 8, where it can be seen that the TCAPLL has a smoothing effect on the solution compared to the stand-alone GPS. This smoothing effect is the result of the complementary error characteristics of the GPS, which have a long term stability navigation solution, and of the INS, which have a short term stability solution.

The TCAPLL provides a better solution; this is shown in Tables 2 and 3 where the standard deviation and mean of the position, velocity and attitude errors are presented. The TCAPLL reduces the position and velocity errors by roughly 70% compared to the stand-alone GPS.

**Figure 9.**
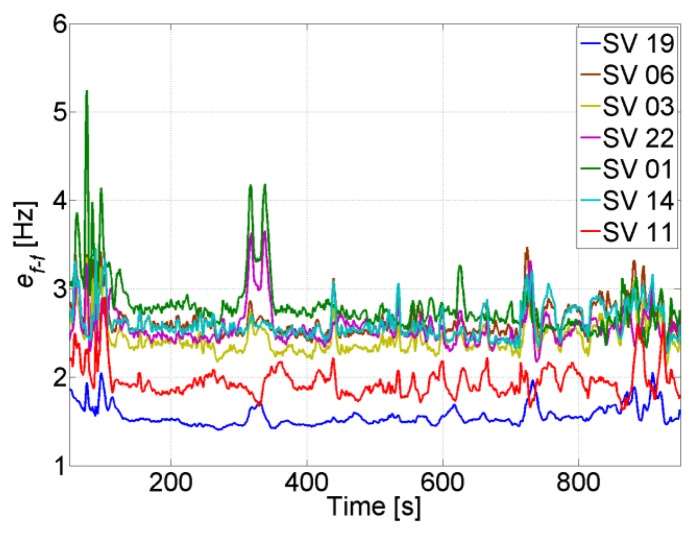
Feed-forward frequency uncertainty in open-sky conditions.

The uncertainty of the aiding frequency of the feed-forward component is shown in Figure 9, where it can be seen that it is smaller than 4 Hz for all satellites. Comparing to Figure 4, the quality of the aiding is good enough for the PLL to maintain tracking.

**Figure 10.**
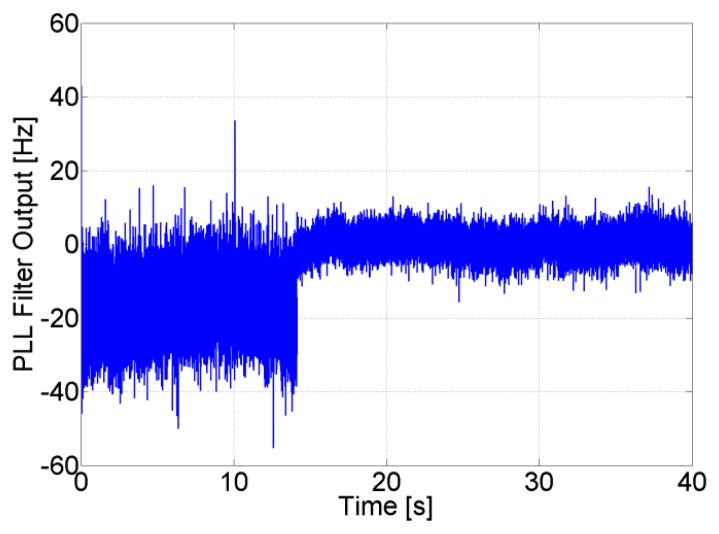
PLL filter output before and after enabling the feed-forward component.

The effect of enabling the feed-forward component on the PLL output is shown in Figure 10. The aiding frequency allows for a reduction of the bandwidth of the PLL. Consequently, after adding the feed-forward frequency to the loop filter output, thermal noise is reduced and the PLL output is centered on zero. In this case the PLL tracks only the induced thermal noise and the error frequency resulting from the aiding.

**Figure 11.**
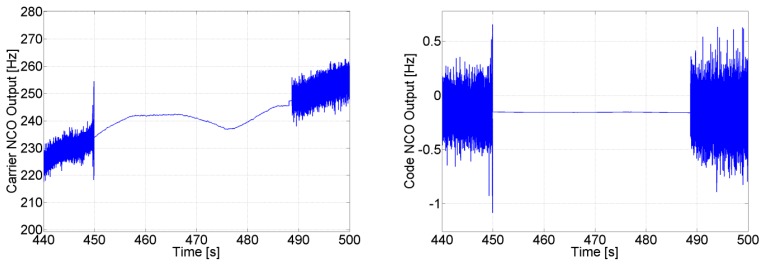
Carrier and code NCOs' behavior during GPS outage.

The advantages of the feed-forward component in avoiding re-acquisition can be seen in Figure 11, where the carrier and code NCOs' output are shown for SV 11. During the GPS outage, the PLL and DLL loops continue tracking using only the feed-forward component, and as soon as the satellite reappears, the tracking loops lock directly to it without the need for re-acquisition. The uncertainty of 2 Hz on the feed-forward component of SV 11 allows to maintain its corresponding generated code phase error uncertainty less than 0.5 chips for roughly 385 s ((*e_f_*_−_*_f_t_Dis_*/SF)<0.5) , allowing a re-tracking without the need for re-acquisition for more than 4 minutes. It is important to mention that with this analysis we are not trying to find an optimal solution for the receiver to re-track a satellite after losing it. Instead we are showing the advantages in terms of re-acquisition and re-tracking that can be obtained when integrating a low quality MEMS-based INS with a very simple GPS receiver having a 2nd order PLL, and provide an assessment of the time during which we can still use the aiding component of the INS for re-tracking, under different GPS conditions as it will be shown later.

**Figure 12.**
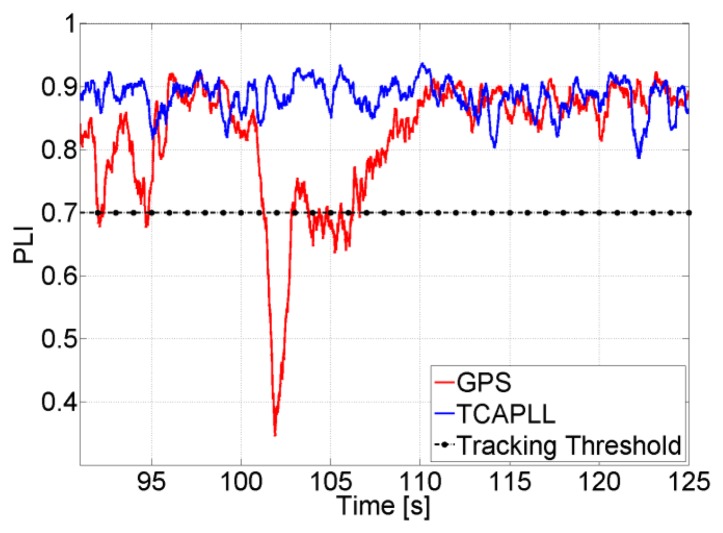
PLI output when *C/N_0_* = 45 dB-Hz, *T_int_* = 1 ms, and *B_n_* = 4 Hz.

As explained before, the feed-forward component allows the PLL to better tolerate the dynamics, and consequently gives the possibility of decreasing its bandwidth. Figure 12 shows the PLI of SV 19 at the first turn during the trajectory with a loop bandwidth of 4 Hz and a high *C/N_0_*. In the case of the stand-alone GPS, the PLL loses lock as soon as there is acceleration and re-tracks again after 5 s. However for the TCAPLL it stays locked all the time.

**Figure 13.**
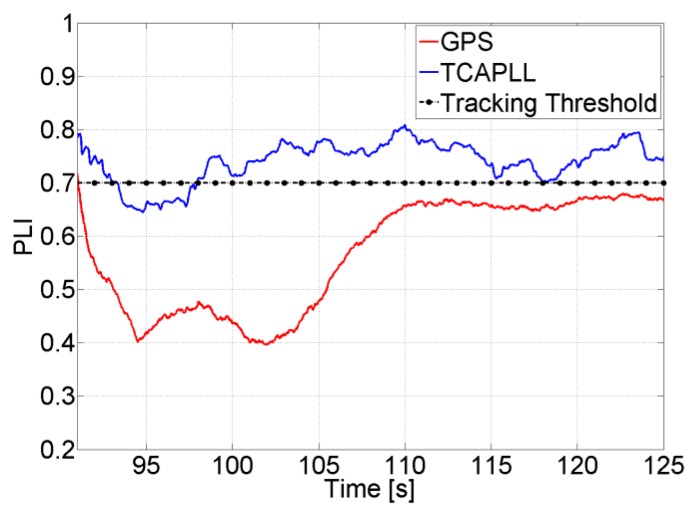
PLI output when *C/N_0_* = 32 dB-Hz, *T_int_* = 5 ms, and *B_n_* = 4 Hz.

The PLI output is shown in Figure 13 for the same scenario but with a lower *C/N_0_* and higher integration time. The PLL loses lock permanently for the stand-alone GPS, whereas the dynamics are compensated with the feed-forward component for the TCAPLL. This shows the importance of the feed-forward component especially in high-dynamic situations where, for the same loop parameters, the sensitivity of the receiver is improved with the TCAPLL in comparison to the GPS.

**Figure 14.**
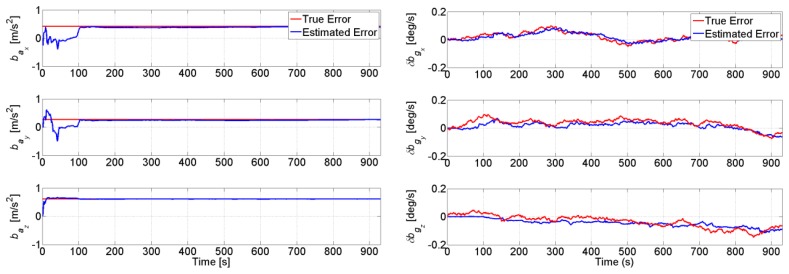
Estimated accelerometer turn-on to turn-on constant bias (**left**) and gyroscope bias drift (**right**).

Figure 14 shows the integration filter estimated turn-on to turn-on constant bias and the bias drift on each axis of the accelerometer and gyroscope, respectively. It can be seen that the TCAPLL EKF estimates correctly the inertial sensor errors. From the left graph, it can be noted that the integration filter needs roughly 100 s to converge. During this time, it is important to have good GPS conditions, otherwise there is a high probability that the integration filter converges to the wrong values. Therefore, when using low-quality MEMS inertial sensors, it is very important to start in good GPS conditions for a certain period of time allowing the integration filter to estimate correctly the error states of the sensors. This duration depends on the quality of the MEMS inertial sensors and the uncertainty on their errors.

In summary, during open-sky conditions the TCAPLL with MEMS inertial sensors provides a better navigation solution than a GPS alone. No re-acquisition is needed once a satellite is lost, as the receiver can continue tracking the satellite based on the aiding information from the INS. In addition, the PLL noise bandwidth can be decreased even in strong dynamic conditions, which provides better sensitivity compared to a GPS receiver with the same loop parameters.

### 4.2. Open Sky with Four SVs

**Figure 15.**
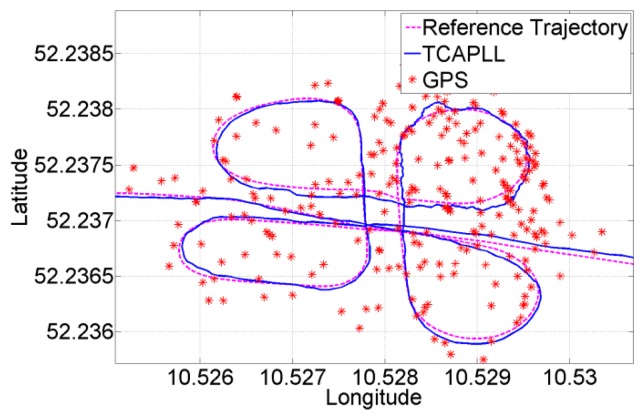
Position solutions when four SVs are visible.

Scenarios where only four satellites are visible represent the minimum condition for a GPS receiver to compute a PVT solution. Therefore it is interesting to assess the performance of the TCAPLL in such conditions. We assume that 500 s after beginning the trajectory, the receiver starts to see only four SVs from the sky plot in Figure 7. The new constellation chosen to be investigated consists of SVs 1, 3, 6 and 19. Its corresponding position solution is shown in Figure 15, and the standard deviation and mean of the position and velocity errors are shown in Table 4. It can be noted that the GPS solution is much noisier than the TCAPLL solution. The latter reduces the position error by 86%, and the velocity error by 92% compared to the GPS.

**Figure 16.**
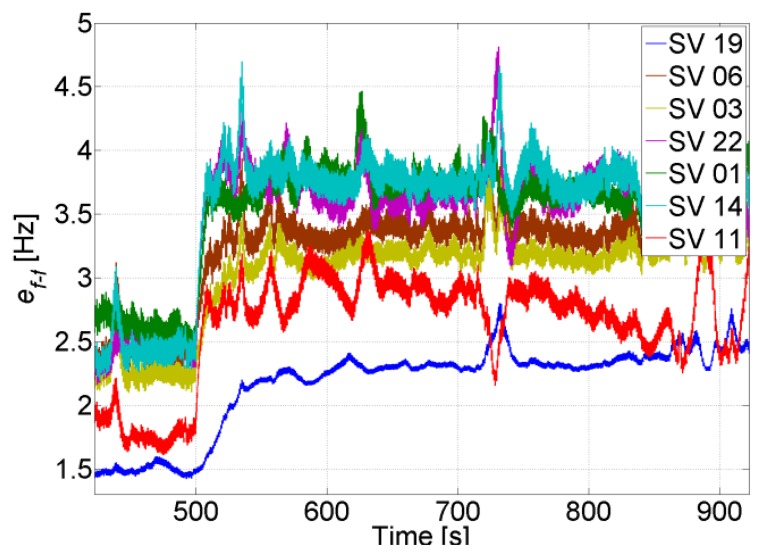
Feed-forward frequency uncertainty when four SVs are in view.

Moreover, Table 4 shows that decreasing the number of visible satellites to four has only a small impact on the velocity solution, which is less sensitive than the position to the change in visibility from seven to four SVs, and from one constellation to another. This is seen in the uncertainty of the aiding frequency of the feed-forward component in Figure 16, where *e_f_*_−_*_f_* is slightly increased. For example, the average frequency error for the non-visible SV 11 is increased to roughly 3 Hz, which allows maintaining a code phase of less than 0.5 chips for 256 s using the feed-forward component only for tracking. If the satellite re-appears within this period, then no re-acquisition is needed.

In summary, when the number of visible satellites decreases to four, the TCAPLL improves the navigation solution compared to the GPS. The position error increase is higher than the velocity error increase, which is less dependent on the number of visible satellites. The quality of Doppler aiding slightly decreases, but is still largely acceptable to maintain robust carrier and code tracking.

### 4.3. Open Sky with Three SVs

**Figure 17.**
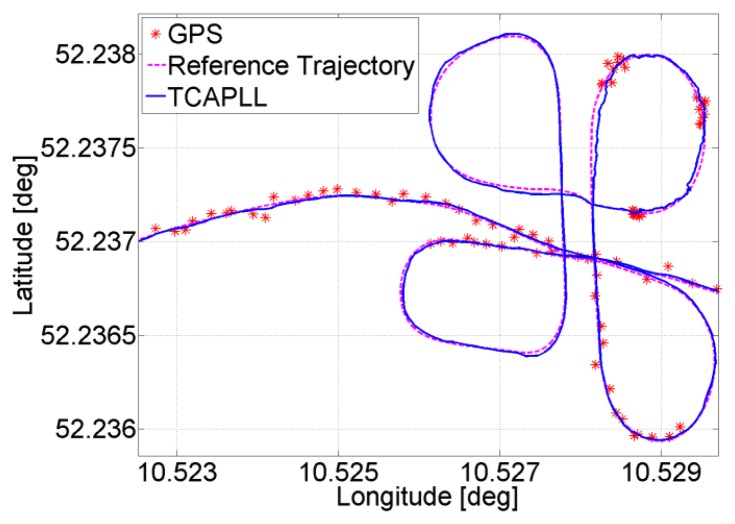
Position solution when three SVs are visible for short periods of time.

A stand-alone GPS receiver cannot provide a 3-D PVT solution when only three SVs are visible. However with tight integration with an INS, this is possible. In this case, the navigation solution starts to depend more on the quality of the INS. In the previous two scenarios, it was shown that once the integration filter converges, the low quality of the MEMS inertial sensor does not have a big impact on the final navigation solution as long as there are continuous GPS updates that correct the INS errors. However, the more the number of visible satellites decreases, the more the final navigation solution depends on the INS. Taking into consideration the sky plot in Figure 7, let's assume that the GPS receiver sees only SVs 19, 6 and 3 during some short periods of time (up to 60 s) and loses visibility of the other satellites. During these periods, the GPS receiver does not provide a PVT solution; however, with the TCAPLL, a navigation solution is computed all the time as shown in Figure 17.

**Figure 18.**
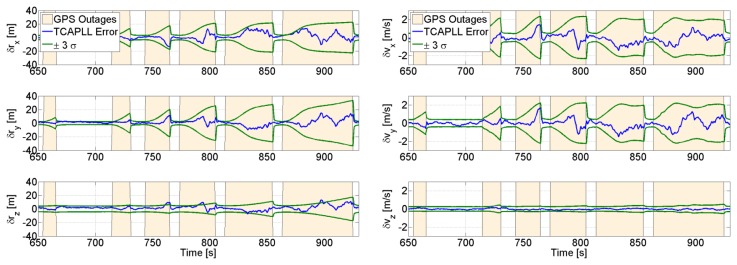
Position (**left**) and velocity (**right**) uncertainties during GPS partial outages.

The uncertainties of the positions and velocities are shown in Figure 18 along with the true errors computed from the reference navigation solution. As soon as the number of satellites drops to three, the uncertainty increases and converges with time. This shows that the integration filter is capable of navigating with only three satellites, but the quality of the navigation solution degrades as expected and becomes more dependent on the INS.

**Figure 19.**
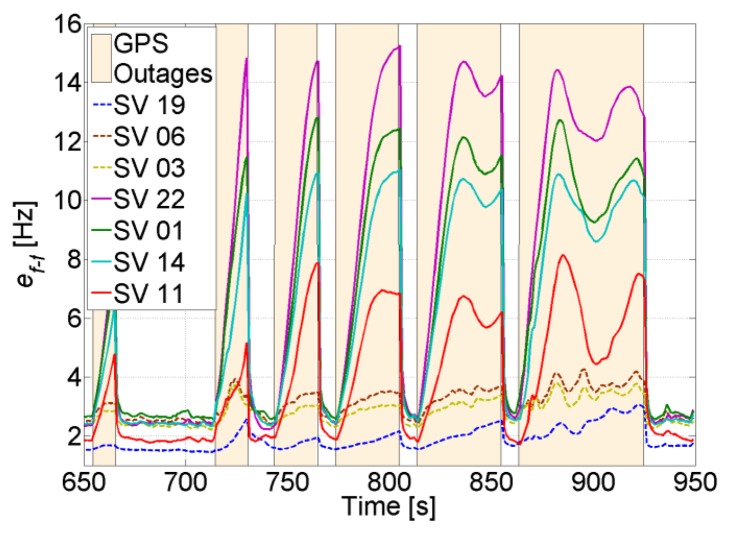
Feed-forward frequency uncertainty when 3 SVs 19, 06 and 03 are in view.

Figure 19 shows the uncertainty of the feed-forward frequency aiding, where the dashed lines refer to the visible satellites. It is clear that the uncertainty for the visible satellites increases less in comparison to the non-visible satellites. Also, the uncertainty increase is inversely proportional to the elevation for the non-visible satellites, *i.e.*, the higher the elevation, the smaller the increase of its uncertainty. This is due to satellite geometry effects on the LOS projection when computing the Doppler frequency uncertainty. Looking back to Figure 4, it can be noted that this increase is still tolerable and will not affect tracking robustness. However, the uncertainties for the non-visible satellites start to be critical, especially if the GPS receiver continue tracking these satellites using the feed-forward component only. For example, the SV 22 uncertainty reaches roughly 15 Hz, which allows maintaining a code phase less than 0.5 chips for almost 51 s without the need for re-acquisition. However if the integration time is 10 ms, then the uncertainty will be higher than the tolerable frequency and consequently re-acquisition will be mandatory.

**Figure 20.**
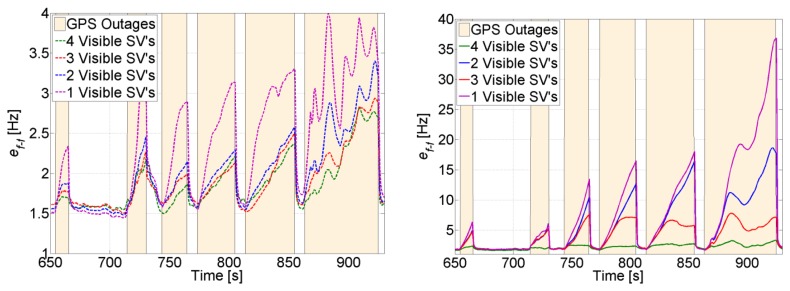
Feed-forward frequency uncertainty for the visible SV 19 (**left**) and the non-visible SV 11 (**right**).

The effect of decreasing the number of visible satellites to four or fewer on the quality of the feed-forward aiding is shown in Figure 20 for a visible and a non-visible satellite, respectively. For instance, SV 19 is always visible and there is a slight increase in its feed-forward uncertainty despite using a low-cost MEMS inertial sensor. Even when SV 19 is the only visible satellite, the frequency error is still tolerable for maintaining carrier and code tracking. However, if we consider SV 11, which is non-visible, the uncertainty increases exponentially, and the effect of the low-quality MEMS sensor is more obvious, especially when the outage time increases. For example, the uncertainty nearly doubles in 60 s every time a satellite is lost. This will put time constraints on the use of the feed-forward component if it is used to re-track the satellite without re-acquiring it. However, if the uncertainty exceeds the maximum tolerable error by the PLL, then a re-acquisition is necessary. In this case, the uncertainty can be used to decrease the frequency search span.

In summary, when the number of visible satellites drops to three, the TCAPLL can still navigate even with low quality MEMS inertial sensors. In this case, the navigation solution quality degrades as it will depend more on the INS. This degradation will converge with time depending on the satellites' geometry, receiver dynamics and the quality of the inertial sensors. Moreover, it was also shown that the position uncertainty is more affected than velocity, and the frequency aiding uncertainty increases slightly for visible satellites, and exponentially for non-visible satellites.

### 4.4. GPS Total Outage

**Figure 21.**
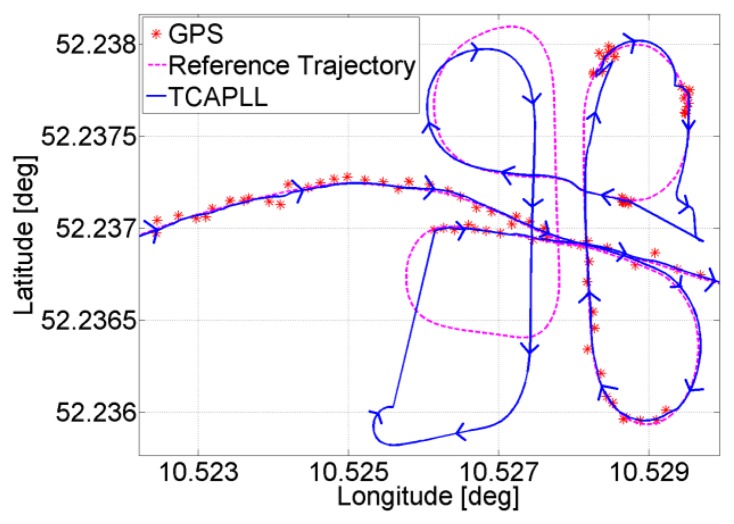
Position solution when GPS total outages occur.

During GPS total outages, the final navigation solution depends solely on the INS. In this case the quality of the MEMS IMU sensors plays an important role in the accuracy of the solution. Moreover, in order to handle long GPS outages, the errors of the sensors should be well modeled and the EKF well-tuned. An example of a position solution during several GPS total outages with different durations is shown in Figure 21. It can be seen that the longer the outage, the higher the drift in the position, which even increases during turns.

**Figure 22.**
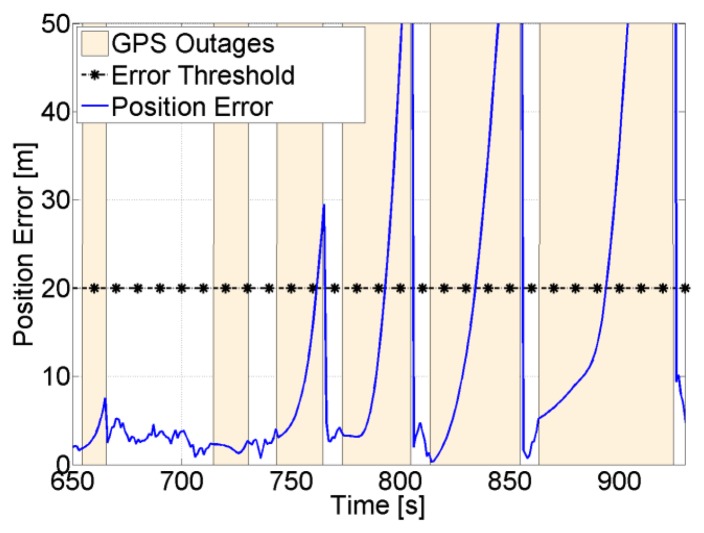
Position uncertainty during GPS total outages.

In order to check how long the navigation solution is valid during GPS outages, the position error is computed and compared to a certain threshold. This threshold will obviously depend on the accuracy requirements. For example, if the maximum allowed position error is set to 20 m, it can be seen from Figure 22 that during the last four outages, the position error takes an average of 22 s to reach 20 m. This means that with typical MEMS inertial sensors such as the one assumed, a maximum GPS total outage of 22 s can be handled. If the quality of the inertial sensors is higher, the navigation time during GPS total outage will be longer, and if the accuracy requirements are higher, the navigation time will be shorter.

**Figure 23.**
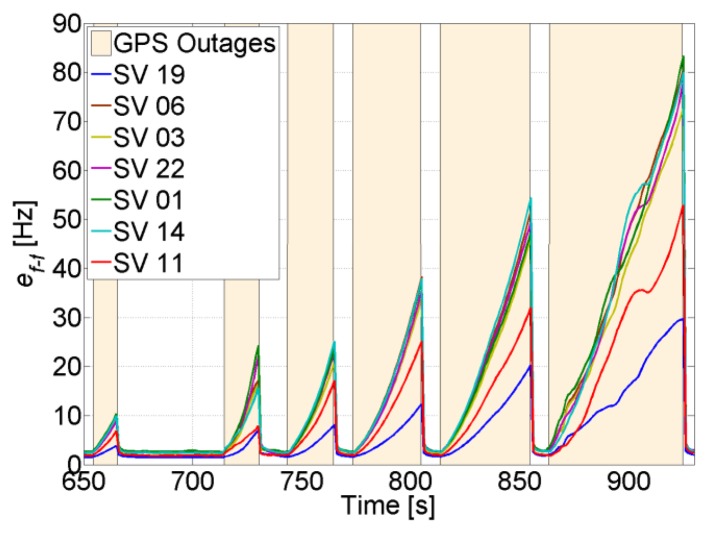
Feed-forward frequency uncertainty during GPS total outages.

The uncertainty of the feed-forward frequency aiding is shown in Figure 23. As expected, the quality decreases during total outages. For the majority of the satellites this will not allow maintaining tracking during outages based on the feed-forward component only, and consequently re-acquisition will be needed to track the satellites once they reappear. For example, for SV 1, the feed-forward uncertainty drifts by roughly 1.33 Hz/s, this means that after 19 s the uncertainty will be 25 Hz. If the satellite does not appear within this time, a PLL with a bandwidth of 4 Hz and integration time of 5 ms will not be able to tolerate this error (see Figure 4), and re-acquisition is needed.

In summary, during GPS total outages, the navigation solution totally depends on the quality of the IMU sensors. In this case, the MEMS inertial sensors are not well suited to handle long outages and can provide a reliable navigation solution for only a very short period of time. Consequently, feed-forward aiding is only temporarily possible, and if the satellite does not reappear before the uncertainty of the aiding drift becomes intolerable, re-acquisition is necessary.

## 5. Field Vehicle Test Measurements

**Figure 24.**
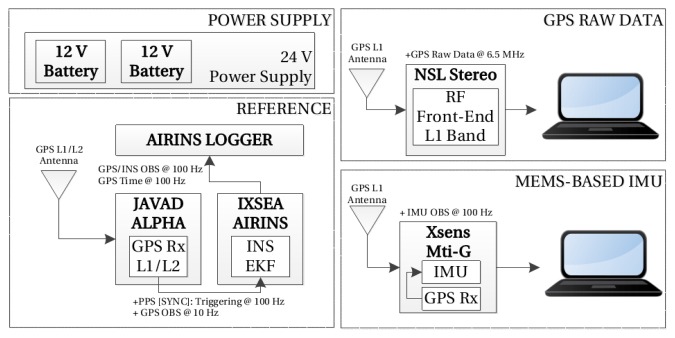
Setup used for the field vehicle test measurements.

**Figure 25.**
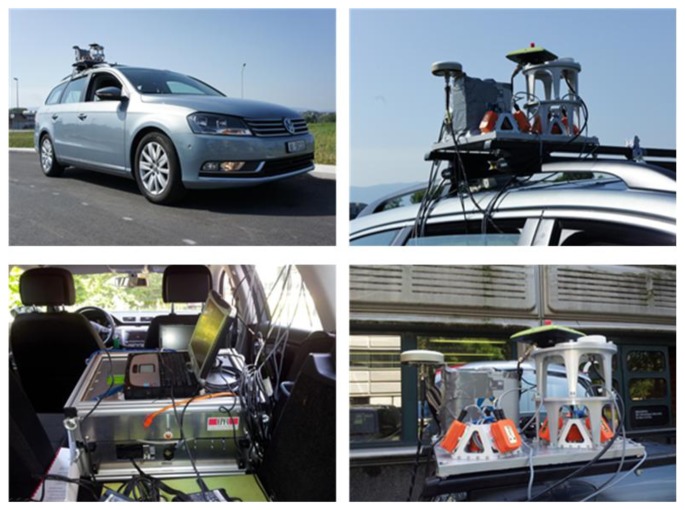
Measurement platform mounted on the test vehicle.

The objective of the measurement campaign is to validate the simulation results obtained in the previous section, and to evaluate the TCAPLL architecture under realistic conditions. The campaign consists of driving a vehicle in urban canyon environments, while capturing GPS signals using an RF front-end, logging IMU measurements using MEMS inertial sensors, and computing a reference trajectory using a tactical grade IMU. The acquired data are post-processed in the laboratory using the TCAPLL architecture to derive a navigation solution. The instrumental setup used is shown in Figure 24 and it is composed of the following parts. The AIRINS navigation grade Georeferencing and Orientation System from IXSEA [27]. The MTi-G Attitude and Heading Reference System (AHRS) from Xsens [28] that contains a MEMS-based IMU, magnetometers, an integrated GPS receiver, a static pressure sensor and a temperature sensor. The Alpha GNSS RTK receiver from JAVAD [29]. The Stereo RF Front-End from NSL [30], which is used to acquire GPS signals in view, then down-convert, digitize and save them on a PC. In addition, multiple GNSS antennas covering the L1/L2 bands are also used. Figure 25 shows the test vehicle mounted with the measurement platform.

The reference navigation solution is computed using INS/GPS integration via optimal forward Kalman filtering and backward smoothing. For that, the IXSEA AIRINS INS is combined with the geodetic-grade Javad Alpha L1/L2 GPS rover receiver (sampling at 10 Hz), and a Topcon Hiper Pro L1/L2 GPS base receiver (sampling at 5 Hz). The carrier-phase GPS observations were double-differenced in post-processing to yield high-precision (cm-level) GPS positioning. These have been combined with the inertial observations in a loosely coupled EKF to obtain a high-precision reference navigation solution [31]. The raw inertial measurements used for the TCAPLL-assisted PLL are obtained from the MTi-G MEMS-based IMU. The parameters used on the modelization of its inertial errors were obtained using the Generalized Method of Wavelet Moments (GMWM) [32,33]. The gyroscope and accelerometer errors on each axis are modeled as a sum of a white noise, a turn-on to turn-on bias and a correlated drift modeled as a Gauss-Markov process. Table 5 shows the values estimated for the error models, which can be considered typical values for low quality MEMS inertial sensors. More details on how these values are obtained can be found in [18].

**Figure 26.**
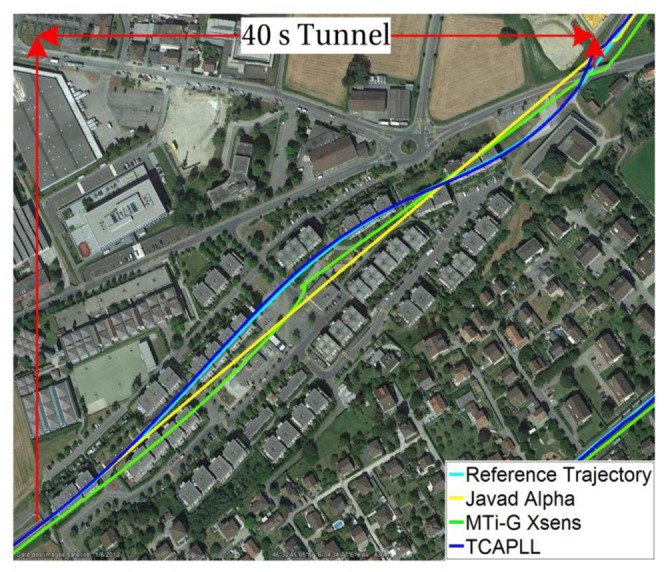
Position solution during the 40 s tunnel in the trajectory from EPFL to Renens center.

**Figure 27.**
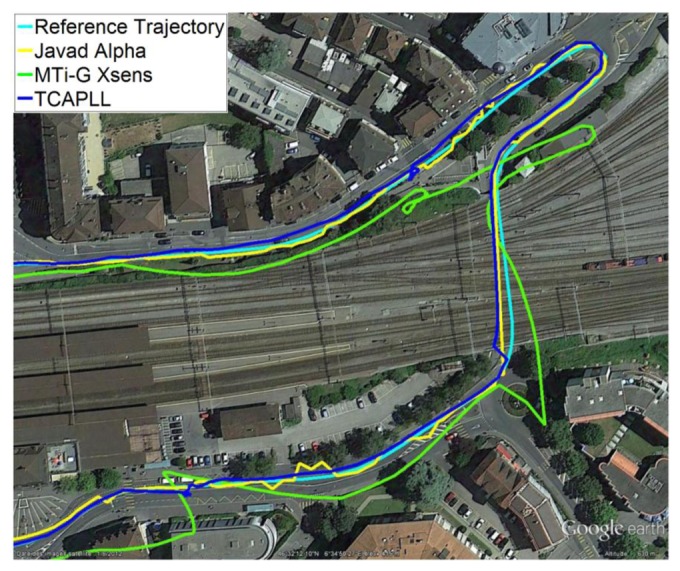
Position solution in Renens center and during a 10 s tunnel under the railway tracks.

**Figure 28.**
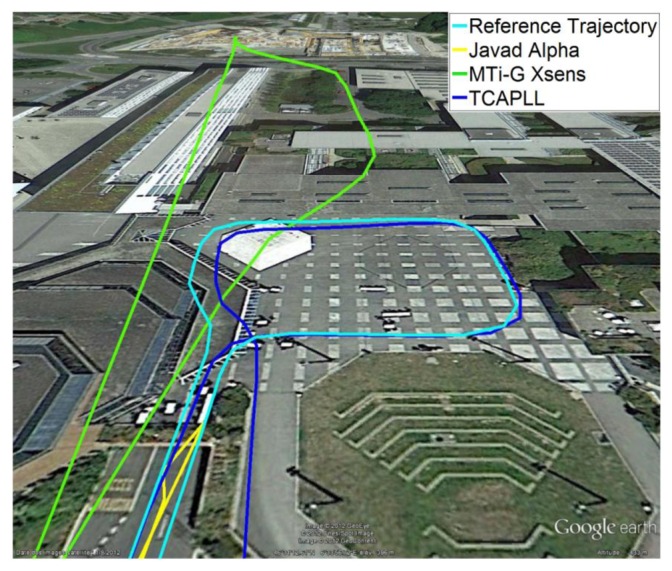
Position solution during the 30 s trajectory in the underground parking structure.

Two trajectories are taken during the field vehicle test measurements: ‘Trajectory 1’ from EPFL to Renens center, with a duration of 920 s including two tunnels having durations of 40 s and 10 s, respectively, and 300 s of driving in urban canyon environments, and ‘Trajectory 2’ on the EPFL campus, with a duration of 430 s including a 30 s drive in an underground parking structure. The INS frequency is set to 100 Hz and the GPS update rate is 1 Hz. The position solution of the TCAPLL in comparison to the reference solution and the solutions from the MTi-G Xsens and the Javad Alpha are plotted in Figures 26, 27 and 28 for different parts of the two trajectories. Figure 26 shows the position solution from Trajectory 1 during the 40 s tunnel. The TCAPLL continues to provide a solution inside the tunnel which closely follows the reference trajectory, and outperforms the MTi-G Xsens solution. The Javad Alpha does not provide a solution in the tunnel as there is a GPS total outage; therefore the yellow path is a straight line from the entrance to the exit of the tunnel. Figure 27 shows the position solution from Trajectory 1 in Renens center and during the 10 s tunnel. Again, the TCAPLL outperforms the MTi-G Xsens solution and provides a position that is very close to the Javad Alpha GPS receiver.

Figure 28 shows the position solution from Trajectory 2 before, during, and after the drive in the underground parking structure. In this case, the TCAPLL continues to provide a position solution, however the drift with time is higher than in the tunnels as the dynamics are different. It can be noted that after each 90° turn, the position solution degrades. Here also, the TCAPLL outperforms the MTi-G Xsens solution. The Javad Alpha receiver does not provide any solution in the parking structure as there is a GPS total outage. The reason the TCAPLL architecture outperforms the MTi-G Xsens is twofold. First, the GMWM provides better modeling of the gyroscope and accelerometer errors, and consequently during GPS total outages when there is total dependence on the inertial measurements, the TCAPLL has far better performance. Second, the integration scheme in the MTi-G is loosely coupled; however, the TCAPLL has tight integration with PLL assistance and a monitoring system to check the quality of the inertial measurements.

**Figure 29.**
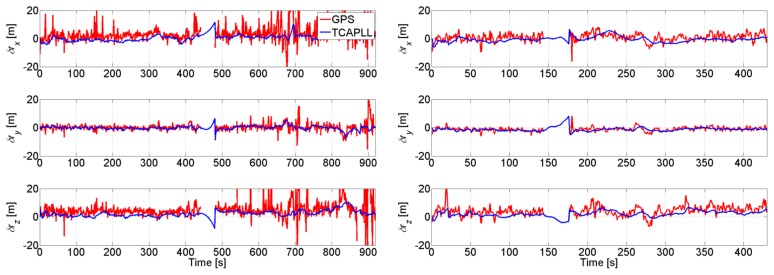
Position error during Trajectory 1 (**left**) and Trajectory 2 (**right**).

The position errors of the TCAPLL during Trajectory 1 and Trajectory 2 are shown in Figure 29, in comparison with the stand-alone GPS solution. The performance of the TCAPLL architecture is better, and this is also shown in Tables 6 and 7 where the standard deviation and mean of the velocity, position, and attitude errors are shown for both trajectories. The TCAPLL velocity error is reduced by 88% and 77%, and the position error by 70% and 42%, respectively, for Trajectory 1 and Trajectory 2. The smaller improvement in Trajectory 2 is due to the fact that the measurements were made mainly in good GPS conditions, for which the stand-alone GPS performs relatively well.

**Figure 30.**
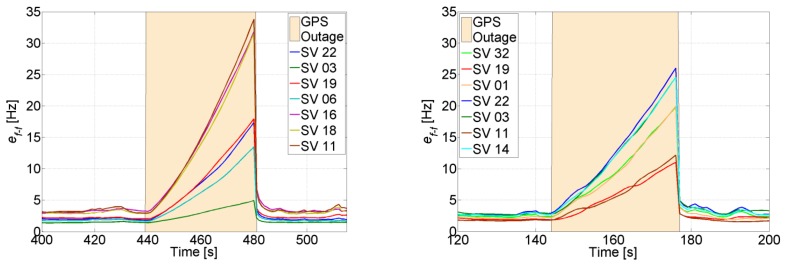
Feed-forward frequency uncertainty during Trajectory 1 (**left**) and Trajectory 2 (**right**).

The uncertainty of the feed-forward component in both trajectories is shown in Figure 30. When there are GPS updates, the uncertainty remains low and in both cases it is less than 5 Hz. This uncertainty is small enough to allow increasing the coherent integration time up to 10 ms without jeopardizing the robustness of the PLL (see Figure 4). However, when there are GPS total outages, the uncertainty increases drastically. The increase varies between each satellite, and is inversely proportional to the satellite elevation, *i.e.*, the higher the elevation of the satellite, the smaller the increase of its feed-forward frequency uncertainty during GPS total outage.

**Figure 31.**
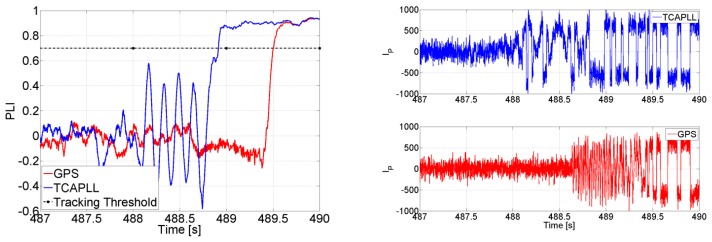
PLI and prompt correlator outputs of SV 22 at the exit of the tunnel in Trajectory 1.

For both trajectories the uncertainties of the feed-forward components during the outages are still acceptable for re-tracking the GPS signals once they reappear, without re-acquisition. For example, for SV 22 in Trajectory 1, the uncertainty reaches 34 Hz in 40 s. This means that at the end of the tunnel the code phase offset of SV 22 is roughly 0.46 chips due to the disabling of the feedback component and the use of only the feed-forward component for tracking. This phase offset is still tolerable by the DLL discriminator, and as can be seen in Figure 31, the TCAPLL re-tracks the signal without re-acquisition. Compared to the stand-alone GPS, for which the receiver has to re-acquire the signal, the TCAPLL is roughly 0.6 s faster in finding the satellites.

**Figure 32.**
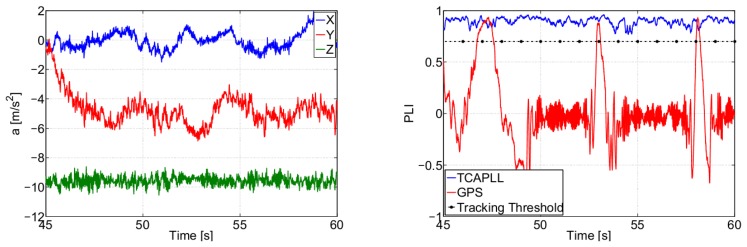
PLI output of SV 19 during high dynamics in Trajectory 1 when *B_n_* = 2 Hz for TCAPLL and 7 Hz for GPS alone.

Finally, Figure 32 shows the PLI output of SV 19 when accelerations of 5 m/s^2^ occur on the y-axis in Trajectory 1. The PLL bandwidth in the TCAPLL is set to 2 Hz and in the stand-alone GPS to 7 Hz. In the TCAPLL, the PLL maintains tracking all the time. However, for the GPS, this is not the case. The PLL loses track as soon as there is acceleration and is unable to track again, and the energy starts to shift between the in-phase and quadrature phase components. This shows the advantage of having a feed-forward component that helps the GPS receiver to cope better with dynamics even when using a 2nd order PLL filter with a bandwidth as low as 2 Hz. The results obtained are comparable to a more sophisticated GPS that would use a higher order loop filter and larger bandwidth.

In summary, the measurements obtained from the vehicle test drive validate the simulation results obtained in the previous section. It has been shown that with the same MEMS inertial sensors, the TCAPLL outperforms the MTi-G Xsens. Also, it provides a performance similar to the Javad Alpha receiver, which is a carrier differential GPS receiver, with the advantage of computing a navigation solution when there are fewer than four visible satellites. In addition, with the TCAPLL, after losing a satellite there is no need for re-acquisition for a certain period of time defined by the uncertainty of the feed-forward component. Moreover, if the satellite reappears after this period, the re-acquisition search span is reduced due to *a priori* information from the aiding frequency component. The analysis provided in this paper proves that with proper initialization and error modeling, the MEMS inertial sensors bring several advantages to the GPS receiver which can be crucial in challenging environments where bad GPS conditions exist.

## 6. Conclusions

In this paper, a GPS/INS Tightly Coupled Assisted PLL (TCAPLL) architecture with a monitoring system was proposed to evaluate the advantages of integrating low-quality MEMS inertial sensors with a GPS receiver. The monitoring system is responsible for checking the quality of measurements coming from the MEMS inertial sensors and the GPS tracking loops in order to ensure robust tracking and accurate navigation. Design and implementation of the TCAPLL were presented and assessment under different scenarios was conducted. It was shown that low-cost sensors, when properly initialized and their errors are well modeled, can bring enhancements to the GPS receiver especially in challenging environments. Also it was shown that they can provide reliable assistance to the PLL to avoid re-acquisition, compensate for receiver dynamics, and hence reduce the loop bandwidths and improve sensitivity. Moreover, it was revealed that low-cost MEMS inertial sensors in the TCAPLL have some limitations, especially for increasing the PLL coherent integration time. Furthermore, they are not well suited when long GPS outages occur, but can provide a navigation solution for short outages depending on the quality of the sensor and the navigation accuracy requirements. Finally, the TCAPLL was evaluated in a field vehicle test with low-quality MEMS inertial sensors. The measurements obtained were in accordance with the simulation results. The TCAPLL outperformed the MTi-G Xsens receiver using the same type of inertial sensors, and in the absence of GPS outages, it provided comparable results to the Javad Alpha GPS receiver in autonomous mode. Finally, it can be concluded that with the continuation of the price decrease and performance increase of MEMS inertial sensors, it is most likely that future mass market GPS receivers will have integrated MEMS-based INS to provide a more reliable, accurate and robust navigation solution.

## Figures and Tables

**Table 1.** Simulated MEMS inertial sensor errors.

	**Gyro**	**Accelerometer**
White noise (*η*) (1–*σ*)	$226\;\text{deg}/\text{h}/\sqrt{\text{Hz}}$	$0.28\;\text{mg}/\sqrt{\text{Hz}}$
1. Turn-on bias (*b*) (1−*σ*)	3,600 deg/h	50 mg
Bias drift GM correlation time (1/*β_d_*) [s]	350	250
Bias drift GM noise *δb* (1–*σ*)	$180\;\text{deg}/\text{h}/\sqrt{\text{Hz}}$	$0.7\;\text{mg}/\sqrt{\text{Hz}}$
2. SF GM correlation time (1/*β_SF_*) [s]	18,000	18,000
SF GM noise (*SF*) (1–*σ*) [PPM]	0.01	0.01

**Table 2.** Standard deviation and mean of the GPS and TCAPLL velocity and position errors.

	**Velocity Error [m/s]**		**Position Error [m]**	

	GPS	TCAPLL	GPS	TCAPLL
*σ*	1.046	0.284	7.998	2.556
mean	0.034	0.017	3.166	0.859

**Table 3.** Standard deviation and mean of the TCAPLL attitude error.

**Error [deg]**	**Roll**	**Pitch**	**Heading**
*σ*	0.237	0.213	1.574
mean	0.094	0.055	0.043

**Table 4.** Standard deviation and mean of the velocity and position errors when only 4 SVs are visible.

	**Velocity Error [m/s]**		**Position Error [m]**	

	GPS	TCAPLL	GPS	TCAPLL
*σ*	5.095	0.376	40.557	5.571
mean	0.567	0.098	20.317	4.615

**Table 5.** Estimated error models of the MTi-G IMU for each axis.

	**Gyro**	**Accelerometer**
White noise (1*σ*)	$240\;\text{deg}/\text{h}/\sqrt{\text{Hz}}$	$2\;\text{mg}/\sqrt{\text{Hz}}$
Turn-on bias (1*σ*)	3260 deg/h	50 mg
GM correlation time [s]	350	30
GM 1*σ* noise	$3\;\text{deg}/\text{h}/\sqrt{\text{Hz}}$	$0.024\;\text{mg}/\sqrt{\text{Hz}}$

**Table 6.** Standard deviation and mean of the GPS and TCAPLL velocity and position errors.

	**Velocity Error [m/s]**				**Position Error [m]**			

	GPS		TCAPLL		GPS		TCAPLL	
Trajectory	1	2	1	2	1	2	1	2
*σ*	3.213	1.995	0.382	0.436	11.831	4.873	3.556	2.787
mean	0.056	0.109	0.096	0.034	6.649	4.505	2.536	2.879

**Table 7.** Standard deviation and mean of the TCAPLL attitude error during Trajectory 1 and Trajectory 2.

**Error [deg]**	**Roll**		**Pitch**		**Heading**	
Trajectory	1	2	1	2	1	2
*σ*	0.159	0.143	0.164	0.157	0.547	1.305
mean	0.096	0.058	0.196	0.185	0.413	1.191
